# ETHNORACIAL DIFFERENCES IN FORMAL AND INFORMAL VOLUNTEERISM AMONG OLDER ADULTS IN THE US: A FOCUS ON ASIAN AMERICANS

**DOI:** 10.1093/geroni/igae098.1853

**Published:** 2024-12-31

**Authors:** Patrick Ho Lam Lai

**Affiliations:** Boston College, Chestnut Hill, Massachusetts, United States

## Abstract

This study questions the assertion that socioeconomic resources are the primary driver behind ethnoracial disparities observed in volunteerism among older adults. Existing literature fails to account for why Asian older adults, with resources comparable to Whites, exhibit lower volunteerism rates. This paper improves upon prior literature by differentiating formal and informal volunteerism and centering Asian American older adults as the reference group to better understand these differences. Utilizing data from the 2019 and 2021 Current Population Survey, bivariate probit models were estimated for formal and informal volunteering. The results indicate that, after accounting for income and education, White individuals have 1.42 times the odds of participating in formal volunteerism compared to Asians, while other ethnoracial groups (Black, Hispanic, American Indian, and Alaskan Native) show 1.09 to 1.31 times the odds of Asians (p <.05). Notably, irrespective of volunteerism type, Asians persist as the least likely to volunteer compared to both White individuals and other ethnoracial groups, underscoring the imperative to investigate other factors such as culture. Implications extend to directing future research toward the cultural underpinnings that may elucidate lower volunteerism rates among Asian older adults. The findings advocate for policy revisions recognizing the intricate interplay between SES and cultural factors. Moreover, social work practices and theoretical models must advance to incorporate cultural considerations, acknowledging the diversity within ethnoracial groups. This nuanced approach is vital in fostering a supportive environment for volunteerism among all older adults, with particular attention to the unique circumstances of Asians.

